# Morphology, Cytotoxicity, and Antimicrobial Activity of Electrospun Polycaprolactone Biomembranes with Gentamicin and Nano-Hydroxyapatite

**DOI:** 10.3390/membranes14010010

**Published:** 2023-12-28

**Authors:** Ioana-Codruta Mirica, Gabriel Furtos, Marioara Moldovan, Doina Prodan, Ioan Petean, Radu-Septimiu Campian, Emoke Pall, Ondine Lucaciu

**Affiliations:** 1 Department of Oral Health, Iuliu Hatieganu University of Medicine and Pharmacy, 400347 Cluj-Napoca, Romania; mirica_codruta@yahoo.com (I.-C.M.); rcampian@email.com (R.-S.C.); ondineluc@yahoo.com (O.L.); 2 Department of Dental Materials, Raluca Ripan, Institute of Research in Chemistry, Babes-Bolyai University, 400294 Cluj-Napoca, Romania; mmarioara2004@yahoo.com (M.M.); doina_prodan@yahoo.com (D.P.); 3 Faculty of Chemistry and Chemical Engineering, Babes-Bolyai University, 400294 Cluj-Napoca, Romania; petean.ioan@gmail.com; 4 Faculty of Veterinary Medicine, University of Agricultural Sciences and Veterinary Medicine, 400372 Cluj-Napoca, Romania; emoke.pall@usamvcluj.ro

**Keywords:** electrospinning, gentamicin, nano-hydroxyapatite, SEM, guided bone regeneration, polycaprolactone

## Abstract

The aim of this research is to develop new nanocomposite membranes (NMs) for guided bone regeneration from polycaprolactone (PCL), with different concentrations of gentamicin sulfate (GEN) and nano-hydroxyapatite (nHAP) through electrospinning. The obtained NMs were characterized for structure through SEM and AFM, which revealed the influence of GEN and nHAP on the fiber diameter. The addition of GEN lowered the fiber diameter, and the addition of nHAP increased the diameter of the fibers. The NMs demonstrated antibacterial properties against *P. aeruginosa*, *S. aureus*, *B. cereus*, and *E. coli* depending on the drug concentration, while being negligibly affected by the nHAP content. NM cytotoxicity assessment, performed once using the MTT assay, revealed no cytotoxicity. The developed NMs could be a promising alternative for guided bone regeneration.

## 1. Introduction

The term guided bone regeneration (GBR) was introduced for the first time in the 1980s as a complement to guided tissue regeneration (GTR) by Nyman and Gottlow [1]. Since then, the use of barrier membranes and bone addition materials in GBR has grown, and it was proven to be useful in periodontal regenerative medicine and implant therapies to increase the formation of new bone tissue [2]. Millions of dental implants are inserted worldwide every year [3], but unfortunately this therapeutic solution is conditioned by the bone supply in all three dimensions, which can be limited [4]. Fortunately, modern medicine can remove this obstacle by reconstructing lost bone tissue using GBR, which currently requires the use of a bone graft along with a barrier membrane [4]. It has been estimated over time that up to 40% of dental implants require GBR before insertion [5]. Several studies have shown that the survival rates of dental implants inserted into sites augmented by the GBR technique are similar to those reported for implants placed in sites with natural bone tissue [6,7]. The survival rate of implants at augmented sites varies between 79% and 100%, with most studies indicating a survival rate of more than 90% at least 1 year after their prosthesis [8].

The electrospinning procedure uses a high voltage to extract the polymer solution from a jet, in ultra-fine fibers, which are deposited on a surface [9]. This electrohydrodynamic process allows us to obtain porous, micro/nano-fibrous structures, from natural or synthetic polymers, including in the polymer matrix (ceramics, metals, and nanocomposites), thus achieving a wide range of multifunctional morphologies [10]. The obtained fibers have a diameter between 50 nm and a few microns and are generally collected in the form of a porous membrane. It has already been shown that the electrospun membranes have the potential to stimulate the function of osteoblastic cells and bone regeneration [9]. The physical, thermal, and mechanical properties of polycaprolactone (PCL) depend on its molecular weight and degree of crystallinity. Its wide use and interesting properties, such as controlled degradability, miscibility with other polymers, and low cost, make it interesting for tissue engineering [11]. The most used synthetic alloplastic materials are hydroxyapatite (HAP) and tri-calcium phosphate (TCP) [12]. Synthetic grafts do not possess osteogenic or osteoinductive properties, but provide minimal immediate structural support, and when applied to healthy bone tissue, osteoid is produced directly on ceramic surfaces in the absence of soft tissue development [13]. Nano-sized HAP (nHAP) particles are associated with a minor level of cytotoxicity in vitro, being characterized by good cell adhesion, stimulating the growth of human osteoblasts [14]. Particles of 20–40 nm play an important role in stimulating biomineralization in vitro [15].

Gentamicin sulfate (GEN) is an aminoglycoside antibiotic largely used for the treatment of many types of infections because it acts against a wide bacterial spectrum. Its disadvantage are its low bioavailability after oral administration, poor cellular penetration, nephrotoxicity, and ototoxicity. Therefore, local delivery of gentamicin is desirable, and innovative preparations based on cement and polymeric beads are already on the market [16].

The novelty of this research is the synthesis, through electrospinning, of new nanomembranes (NMs) from PCL containing 0, 10, 15 wt.% nHAP and/or 0.5, 1 wt.% GEN. The NMs were evaluated for morphology, antibacterial activity, and cytotoxicity.

## 2. Material and Methods

### 2.1. Materials

PCL with molecular weight (M.W.) 80,000 g·mol^−1^, methanol, chloroform, GEN, Mueller Hinton Agar, 12-well plates, 96-well plates, and Petri dishes were acquired from Sigma-Aldrich. Dimethyl sulfoxide (DMSO) was obtained from Honeywell, Fluka, Seelze, Germany. The bacterial strains *E. coli* ATCC 25922, *S. aureus* ATCC, *B. cereus* ATCC 11778, and *P. aeruginosa* ATCC 10,145 were obtained from the American Type Culture Collection (ATCC). The culture medium DMEM/F12 and the culture medium supplemented with 10% fetal calf serum, streptomycin, and penicillin were acquired from Gibco LifeTechnologies, Paisley, UK. All commercial materials were used without further purification.

#### 2.1.1. Synthesis of nHAP and Preparation of the Electrospun NMs

The synthesis of nHAP was described in a previous work [17]. The preparation of the ceramic/drug/polymer solution took place by dissolving GEN in a solvent solution of methanol/chloroform (3:1), stirring for 5 h with a magnetic bar, and then sonicating for 30 min. After adding PCL, the agitation process was continued for the same period and in the same conditions. The nHAP nanoparticles were added to the solution, which was sonicated using ELMA Elmasonic P70H (Elma Electronic Inc., Fremont, CA, USA) for 30 min, power 90%, frequency 80 KHz, at room temperature followed by stirring for 14 h and sonicating again for 30 min at the end. For all obtained NMs, 2 g of PCL was used in order to obtain similar thickness for all the NMs. The electrospinnning process was carried out with an experimental machine from Raluca Ripan, Institute of Research in Chemistry, Cluj-Napoca, Romania, at the following parameters: the distance between the tip of the needle and the collector was 31 cm, the flow rate was 2.5 mL/h, and the voltage was 15–25 kv. The obtained NMs (Table 1) were stored in a desiccator for 48 h before running the tests.

#### 2.1.2. Morphological Characterization of the Obtained NMs

The morphology of the NMs was analyzed by SEM (SEM Inspect S, FEI, The Netherlands) using a high vacuum, and the fiber diameter from the SEM image was evaluated using Image J software. The mean diameter and the standard deviation (SD) were calculated after collecting the data, and then graphs were generated.

Atomic force microscopy (AFM) investigation was run by cutting a small piece from each of the electrospun NMs and mounting it on the specimen holder with double adhesive tape. AFM was effectuated on a JEOL JSPM 4210 Scanning Probe Microscope, produced by JEOL, Japan, Tokyo. The cantilevers used for surface investigation are from the NSC 15 series produced by MicroMasch, Estonia, Tallinn, having a resonant frequency of 325 kHz and a force constant of 40 N/m. The topographic images were scanned at an area of 10 μm × 10 μm for the fine microstructure observation and at 2.5 μm × 2.5 μm for the nanostructural aspects. The scan rate ranges from 1.5 to 3 Hz depending on the scanned area. The images were analyzed using the specialized software JEOL WIN SPM 2.0 produced by JEOL, Japan, Tokyo. Surface roughness parameters *Ra* and *Rq* were measured for each image. In order to obtain a proper statistical average, at least three different macroscopic areas for each sample were scanned.

*Ra* represents the arithmetic average of the profile height and is described by Equation (1), and *Rq* represents the root mean square of the profile height and is described by Equation (2):

(1) $$Ra=\frac{1}{l_{r}}\int_{0}^{l_{r}}\left|z(x)\right|dx$$

and

(2) $$Rq=\sqrt{\frac{1}{l_{r}}\int_{0}^{l_{r}}\left|z{\left(x\right)}^{2}\right|dx}$$

where *l* is the profile length and *z* is the height at *x* point. Both *Ra* and *Rq* are important for various research applications [18,19].

#### 2.1.3. Antibacterial Activity of the Obtained NMs

The GEN-loaded NMs were tested for the antibacterial effect with a disk diffusion assay on *E. coli*, *S. aureus*, *B. cereus*, and *P. aeruginosa* by inoculating the surface of a Mueller Hinton Petri dish with 1 mL of bacterial suspension at a density of 0.5 McFarland. The BM disks (5 mm diameter, 0.2 mm thickness) with different concentrations of GEN and nHAP were placed on the inoculated solid media and incubated for 24 h at 37 °C. The tests were run in triplicate.

#### 2.1.4. Cytotoxicity

The samples of the NMs were sterilized by β irradiation at 25 Gy for 15 min before incubation for 1 day in cell culture medium (DMEM supplemented with 10% fetal bovine serum), penicillin (100 U/mL), and streptomycin (100 µg/mL) at a concentration of 0.1 g/mL according to ISO 10993 [20]. The collected media were directly used for the MTT assay, which utilized human fibroblasts. Then, 100 mL of fibroblast cells was added to each well of a 96-well plate at a density of 10^5^ cells/mL and exposed for 24 h to 80 µL collected media during an incubation at 37 °C in the presence of 5% CO_2_. Untreated cells were used as a control. The culture medium was removed and 100 μL of MTT solution (1 mg/mL) was added and incubated for 3 h at 37 °C. DMSO solution was used to dissolve the formazan and the chromogenic reaction was evaluated with a BioTekSynergy 2 microplate reader (Winooski, VT, USA) at 450 nm. Each experimental condition was performed in triplicate. The calculation of the results was carried out according to Equation (3).

(3) $$Cell\;viability\;(\%)\;=\;\frac{Absorbance\;of\;the\;NM-Absorbance\;of\;the\;medium\;control}{Absorbance\;of\;the\;control-Absorbance\;of\;medium\;control}\times 100$$

#### 2.1.5. Statistical Analyses

The obtained results were processed to calculate the average value and the standard deviation and to generate graphs. The fiber diameter, cytotoxicity, and antibacterial activity values were statistically analyzed with a *t* test, with the level of significance set at *p* < 0.05.

## 3. Results

**Figure 1:**
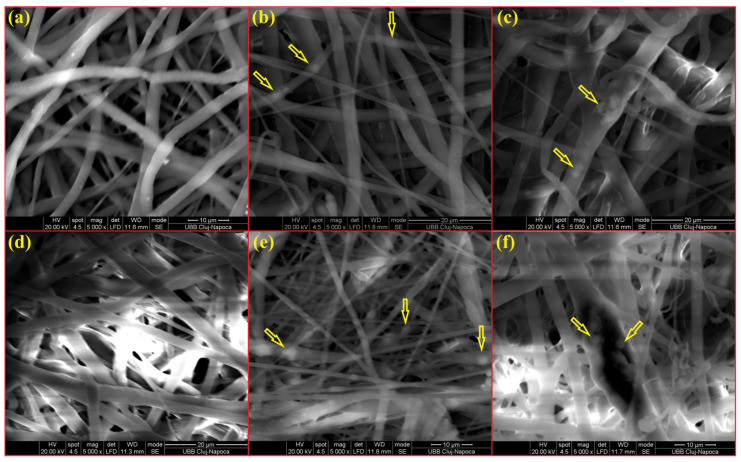
SEM micrographs of NMs: (**a**) M_0_, (**b**) M_1_, (**c**) M_3_, (**d**) M_4_, (**e**) M_5_, and (**f**) M_6_.

**Figure 2:**
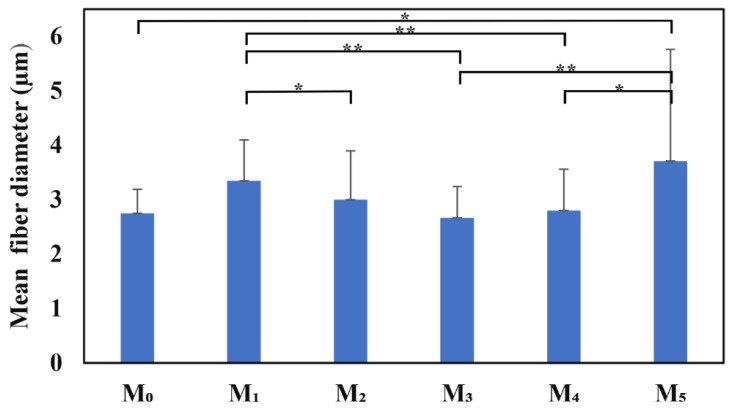
Fiber diameter from SEM images of NMs (* *p* < 0.05; ** *p* < 0.01).

**Figure 3:**
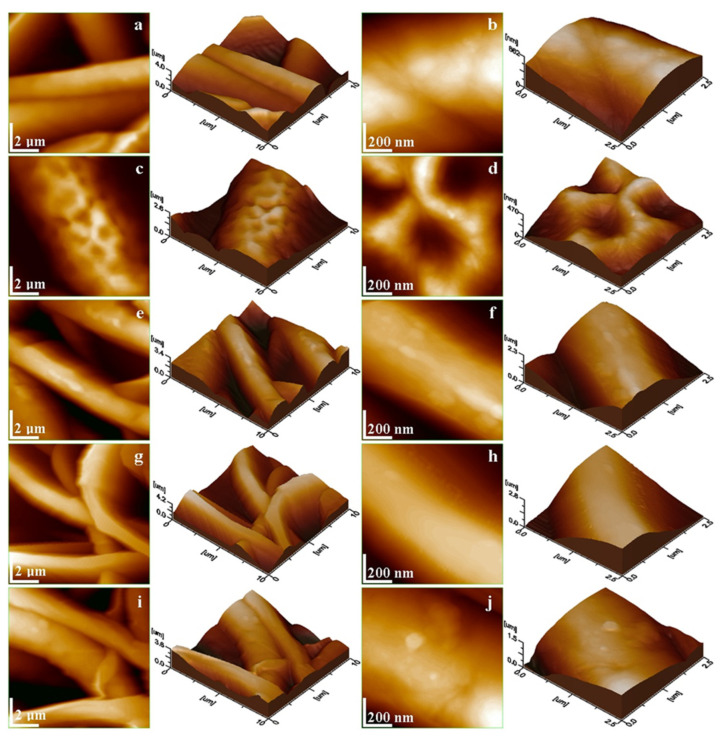
AFM topographic images of the fibers within electrospun fine microstructure: (**a**) M_0_, (**c**) M_1_, (**e**) M_2_, (**g**) M_3_, (**i**) M_4_ and nanostructure: (**b**) M_0_, (**d**) M_1_, (**f**) M_2_, (**h**) M_3_, (**j**) M_4_. Three-dimensional profiles are given at the right side of each topographic image.

**Figure 4:**
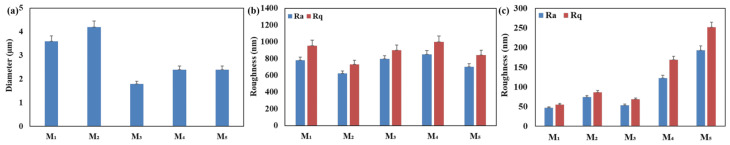
Values measured on AFM images: (**a**) fiber mean diameter, (**b**) fine microstructure roughness, and (**c**) nanostructure roughness.

**Figure 5:**
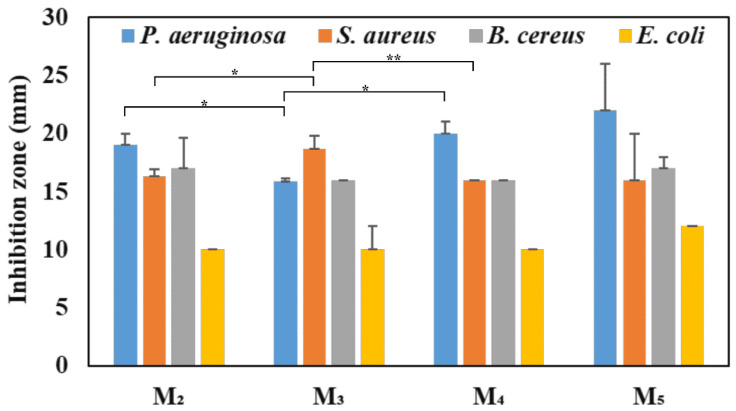
Inhibition zones of the GEN-loaded NMs on *P. aeruginosa*, *S. aureus*, *B. cereus*, and E. coli (* *p* < 0.05; ** *p* < 0.05).

**Figure 6:**
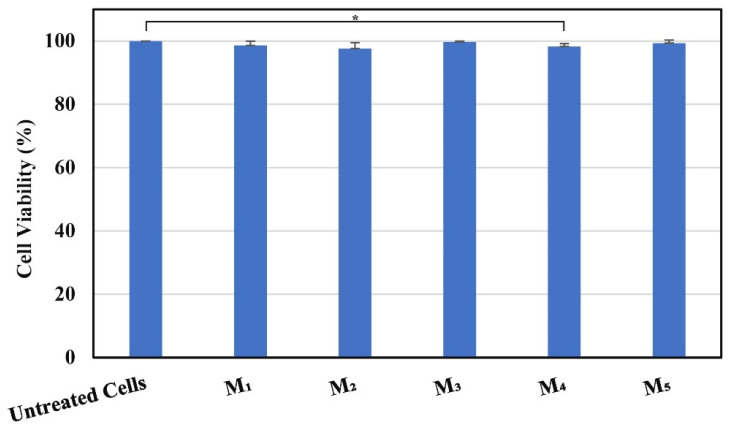
Cell viability after 1 day exposure with NMs, investigated on human fibroblasts with an MTT assay (* p-value < 0.05).

SEM images of NMs show a porous structure with the size of the fibers ranging from 2.67 to 3.71 µm (Figure 1 and Figure 2). The AFM image in Figure 3 shows a mean diameter of fiber around about 3.6 µm and a relatively smooth surface. Figure 4a shows a mean diameter of fiber about 1.8 µm. Figure 3f shows clusters with lengths in the range of 200–600 nm and width about 120 nm; M_5_ shows 2.4 µm. In Figure 3j, we observe clusters ranging from 100 to 200 nm diameter. The composition of each sample affects the fiber surface roughness that was measured with AFM on the top of the visualized fibers to obtain the proper characteristic value without any influence from their relative topographic positions within the sample microstructure. We screened the antibacterial activities of the NMs loaded with GEN using a disk diffusion assay (Figure S1 and Figure 5). The growth inhibition zones were between 10 and 22 mm (Figure S1 and Figure 5). The results of the MTT assay performed against human fibroblasts on 1 day show a cell viability between 97.69% and 99.77% (Figure 6).

## 4. Discussion

SEM investigation of the obtained NMs (Table 1) showed typical, randomly aligned bead-free electrospun fibers in different layers, with an interconnected porosity (Figure 1). The yellow arrows in Figure 1b,c,e,f show white dots inside of fibers associated with nHAP particles. M5 had the fibers with the highest diameter, which could be explained by the highest amount of nHAp (15 wt.%). The M_1_ sample containing only PCL + 10% nHAP formed a dense and interlaced fiber network, as observed by SEM microscopy. The AFM image in Figure 3a reveals its fine microstructure, showing the topography of four representative fibers. Their mean diameter is about 3.6 µm, and they present a smooth and uniform surface. The lower side of Figure 3a evidences the marginal side of a bigger fiber intersection. The nanostructural aspect is evidenced by the topographic image in Figure 3b, taken on the fiber surface. The surface is relatively smooth and the PCL-embedded nHAP particles are situated in the outermost layer (about 90 nm); the diameter is slightly increased by the polymer coating of the nHAP particles. The material inside the fiber is very compact and has some submicron clusters with diameters of about 400 nm. The M_2_ sample contains PCL with 0.5 wt.% GEN (Figure 3c), featuring vigorous fibers of about 4.2 µm in diameter (the mean value is represented in Figure 4a). The surface topography is affected by regular pits of 250 nm diameter and 200 nm depth. These are induced by the GEN functionalization of the material. The pits have a regular nanostructural pattern with polymer crystallites ranging from 60 to 70 nm (Figure 3d).

Adding 0.5 wt.% GEN to the PCL with 10 wt.% nHAP induces slightly smaller fibers of M_3_ with a mean diameter of about 1.8 µm (Figure 4a), with a compact microstructure of internal submicron clusters prolonged along the extrusion direction (Figure 3e). Three main fibers appear interlaced, with the other two situated on the lower plane of the observation field. The observation of a single fiber at the nanostructural level (Figure 3f) better reveals the prolonged clusters, which range in length from 200 to 600 nm and are about 120 nm wide. They are mostly caused by the material flowing during electrospinning through the nozzle. However, well-dispersed nHAP particles well embedded into the polymer matrix are observed in the outermost layer of the fiber with a diameter of 70 nm.

M_4_ contains 10 wt.% nHAP and a GEN content increased to 1 wt.%. Fact induces topographical changes within the fibers (Figure 3g). The observation field evidences the multiple intercalations of four principal fibers and two situated on the lower plan. These are more vigorous and dense, having a mean diameter of about 2.4 µm and a very smooth surface without any submicron clusters. The nanostructural aspect is also very smooth (Figure 3h), whereas the fiber sides are more visible than the nHAP particles embedded into the outermost layer of PCL. Their diameter with polymeric coating is around 75 nm. Both the fine microstructural aspects and nanostructure of M_4_ fibers indicate a synergistic equilibrium between nHAP and GEN content.

Furthermore, M_5_ has an increased amount of 15 wt.% nHAP related to 1 wt.% GEN. The extra filler amount introduced in this sample affects the fiber diameter, which is still 2.4 µm, less, but again induces the apparition of submicron clusters developed in the inner material (Figure 3i). There is a main fiber that appears slightly bigger than the other two, which are interconnected by local adhesion necks. The nanostructure topography (Figure 3j) reveals rounded submicron clusters ranging from 100 to 200 nm in diameter. The outermost layer of the fiber clearly presents well-individualized nHAP particles well embedded into the polymer matrix with a diameter of about 82 nm.

The fine microstructure roughness (Figure 4b) depends only on the fibers’ positions and their interlacing network, and did not present any correlation with the material composition. Thus, Figure 4c shows that M_1_ has the lowest roughness value among all investigated samples, besides the observation of internal submicron clusters. The M_2_ sample containing only PCL and GEN has a greater roughness than the M_1_ sample due to the occurrence of superficial pits. Furthermore, Figure 4c shows that addition of 0.5 wt.% GEN to the mixture of PCL + 10 wt.% nHAP induces a slow increase in roughness of M_3_ compared to M_1_. This indicates that GEN is able to increase fiber roughness. This is sustained by a significant increase in roughness after increasing the amount of GEN to 1 wt.% in M_4_. Adding 5 wt.% more nHAP filler to the composition with 1 wt.% GEN in M_5_ induces another significant roughness increase. M_0_ and M_1_ were tested (Figure S1) but do not appear in Figure 5 because they did not show any antibacterial activity. The largest growth inhibition was registered for *P. aeruginosa* between 16 and 22 mm and the smallest for *E. coli* between 10 and 12 mm (Figure 5). The inhibition zone for *S. aureus* registered between 16 and 19 mm, and for *B. cereus* between 16 and 17 mm (Figure 5). The only statistical difference is between the control (untreated cells) and M_4_. It appears that adding GEN together with nHAP lowers the cell viability to 98.30%, but adding 15% nHAP at the same concentration of GEN (1 wt.%) increases the cell viability to 99.38%.

Over time, different research groups have focused on the regeneration of the bone, developing different types of materials which should speed and improve the healing period, choosing nHAP as the inorganic filler [17,21,22]; besides optimal results in vitro, it also shows promising results in vivo [23]. Some studies have preferred electrospinning as the method for obtaining materials because it can facilitate the acquisition of a typical electrospun matrix similar to the extracellular matrix [10]. As in other studies [17,24,25], SEM images of the NMs revealed a typical electrospun matrix. Electrospun fibers are one of the most widely proven platforms for growing soft and hard tissues, because they create a perfect micro-environment for cell adhesion, proliferation, and differentiation. Also, nanoscaffolds are proven to aid in the growth of different types of cells and tissues [26]. Our results showed that the fiber diameter is affected, as indicated by the GEN/nHAP content, also shown in previous research [27]. A structure made of fine fibers contributes to the increase in the surface-to-volume ratio and shapes pores with different sizes, a fact considered essential for cellular growth in vitro and in vivo, being directly involved in oxygen and nutrient transport to cells [28]. AFM investigation showed different roughness values, depending on the quantity of nHAP in the NMs. Data in the literature show that slightly increased roughness of the biomaterial surface facilitates better proliferation of cultured cells such as osteoblasts [29,30], and increases the antibacterial effect by preventing dangerous microorganisms’ adhesion to their surface [31,32]. Thus, the significant roughness increase associated with good dispersion of the nHAP within the fiber materials, and subsequently within their surface, along with GEN loading reveals a synergistic effect on the microstructure, which should be correlated with the samples’ antibacterial effect. Postoperative infections are one of the most serious complications associated with bone grafts, *S. aureus* [33] being the most common bacterial strain Other bacterial strains of principal concern are *E. coli* [34] and *P. aeruginosa*, which is believed to be the most common Gram-negative bacteria in nosocomial infections [35] because it produces a compound named Exotoxin A, which causes local tissue damage [36]. The obtained result for *S. aureus* (Figure 5) highlights the fact that the addition of nHAP lowers the antibacterial activity of a material, as proven before [24]. Other researchers state that an inhibition zone larger than 15 mm is regarded as a good predictor of efficient antibacterial activity [37,38]. The obtained high cell viability is similar to another study [39], which used a comparable composition of materials. In our study, adding 15 wt.% nHAP at 1 wt.% GEN increased the cell viability to 99.38%, proving the ability of nHAP to stimulate the cell growth [14]. NMs loaded with nHAP showed a cell viability over 95%, as in previous research [40]. As established in ISO 10993 [20], any material is cytotoxic if the cell viability is less than 70%. From the above observation, it can be said that the obtained NMs are nontoxic, cytocompatible, and ideal for biomedical application [40].

## 5. Conclusions

In this study, several NMs were obtained with the following composition: PCL; PCL-10%nHAP; PCL-10%nHAP-0.5%GEN; PCL-1%GEN; PCL-10%nHAP-1%GEN; and PCL-15%nHAP-1%GEN. The typical electrospun structure of the NMs was confirmed by SEM and AFM analyses, as well as the influence of the GEN/nHAP content on the fiber diameter. The addition of GEN lowered the fiber diameter, and the addition of nHAP increased the diameter of the fibers. The antibacterial properties were proven in different bacterial strains: *P. aeruginosa*, *S. aureus*, *B. cereus*, and *E. coli*. The lack of cytotoxicity was highlighted through the MTT assay run at 24 h on human fibroblasts. The obtained results encourage us to run further in vitro and in vivo studies to prove that the developed NMs could represent an alternative to the membranes used for GBR today.

## Figures and Tables

**Table 1:** The obtained NMs.

No.	Code	Composition of the NMs		
		PCL (wt.%)	nHAP (wt.%)	GEN (wt.%)
1	M_0_	100	0	0
2	M_1_	90	10	0
3	M_2_	89.5	10	0.5
4	M_3_	99	0	1
5	M_4_	89	10	1
6	M_5_	84	15	1

Note: The quantity of GEN in samples used was 0.01117 g M_2_; 0.02020 g M_3_; 0.02247 g M_4_; and 0.02381 g M_5_. For obtaining each material, 3.75 mL chloroform and 11.25 mL methanol were used.

## Data Availability

The data that support the findings of this study are contained within the article.

## Supplementary Materials

The following supporting information can be downloaded at: https://www.mdpi.com/article/10.3390/membranes14010010/s1, Figure S1: Disk diffusion assay on: (a) *S. aureus*; (b) *P. aeruginosa*; (c) *E. coli* and (d) *B. cereus*.
