# Supplementary material for: Morphology, Cytotoxicity, and Antimicrobial Activity of Electrospun Polycaprolactone Biomembranes with Gentamicin and Nano-Hydroxyapatite
Source: Membranes (Basel). 2023 Dec 28;14(1):10. doi: 10.3390/membranes14010010 (PMC10819002; doi:10.3390/membranes14010010)
Supplement: Supplementary file 1 [file membranes-14-00010-s001.zip › membranes-2742750-supplementary.pdf]

Supporting Information

for Membranes

Morphology, Cytotoxicity, and Antimicrobial Activity of Electrospun Polycaprolactone Biomembranes with Gentamicin and Nano-Hydroxyapatite

Ioana-Codruta Mirica[1], Gabriel Furtos[2,*], Marioara Moldovan[2], Doina Prodan[2], Ioan Petean[3], Radu-Septimiu Campian[1], Emoke Pall[4], Ondine Lucaciu[1]

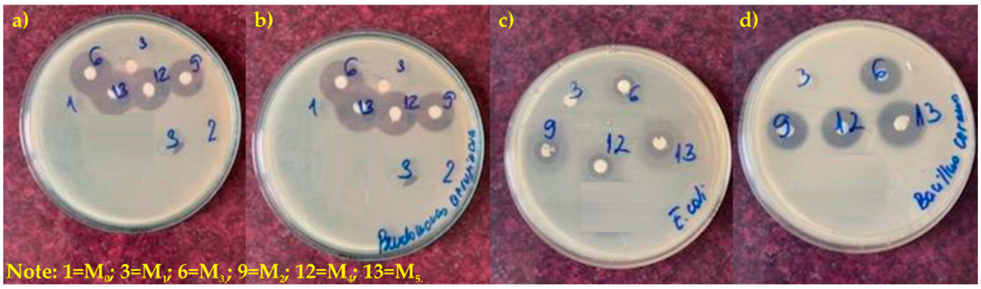

Figure S1. Disk diffusion assay on: a) *S. aureus;* b) *P. aeruginosa*; c) *E. Coli* and d) *B. cereus.*
